# Transfusion of Human Platelets Treated with Mirasol Pathogen Reduction Technology Does Not Induce Acute Lung Injury in Mice

**DOI:** 10.1371/journal.pone.0133022

**Published:** 2015-07-15

**Authors:** Axelle Caudrillier, Beñat Mallavia, Lindsay Rouse, Susanne Marschner, Mark R. Looney

**Affiliations:** 1 Department of Medicine, University of California San Francisco, San Francisco, California, United States of America; 2 Terumo BCT, Inc., Lakewood, Colorado, United States of America; 3 Department of Laboratory Medicine, University of California San Francisco, San Francisco, California, United States of America; Emory University/Georgia Insititute of Technology, UNITED STATES

## Abstract

Pathogen reduction technology (PRT) has been developed in an effort to make the blood supply safer, but there is controversy as to whether it may induce structural or functional changes to platelets that could lead to acute lung injury after transfusion. In this study, we used a commercial PRT system to treat human platelets that were then transfused into immunodeficient mice, and the development of acute lung injury was determined. P-selectin expression was higher in the Mirasol PRT-treated platelets compared to control platelets on storage day 5, but not storage day 1. Transfusion of control vs. Mirasol PRT-treated platelets (day 5 of storage, 10^9^ platelets per mouse) into NOD/SCID mice did not result in lung injury, however transfusion of storage day 5 platelets treated with thrombin receptor-activating peptide increased both extravascular lung water and lung vascular permeability. Transfusion of day 1 platelets did not produce lung injury in any group, and LPS priming 24 hours before transfusion had no effect on lung injury. In a model of transfusion-related acute lung injury, NOD/SCID mice were susceptible to acute lung injury when challenged with H-2Kd monoclonal antibody vs. isotype control antibody. Using lung intravital microscopy, we did not detect a difference in the dynamic retention of platelets in the lung circulation in control vs. Mirasol PRT-treated groups. In conclusion, Mirasol PRT produced an increase in P-selectin expression that is storage-dependent, but transfusion of human platelets treated with Mirasol PRT into immunodeficient mice did not result in greater platelet retention in the lungs or the development of acute lung injury.

## Introduction

The safety of the blood supply has progressively improved since the advent of modern transfusion therapy in the first half of the 20^th^ century. The use of nucleic acid amplification testing for hepatitis B, hepatitis C, and the human immunodeficiency virus have substantially reduced the transmission risk to 1:1–10 million [1]. However, substantial residual risk of transmission remains for known and emerging bacterial, viral, and parasitic infections that can have devastating consequences to the recipient. Platelets are particularly a concern for the transmission of bacterial infections, since platelets are stored at room temperature prior to transfusion. Another concern is the transfusion of fresh, warm whole blood in the military when there is not enough time or resources to screen the blood for pathogens [2].

To address this problem, there have been a variety of pathogen-reduction technologies (PRT) that have been developed to deactivate pathogens prior to transfusion therapy [3]. These technologies efficiently target possible pathogens, but there is concern that donor cells (red blood cells, platelets) can be damaged in the process. In a prospective study in which human platelet units were treated with a psoralen-based PRT, there was an excess of patients in the PRT arm that developed the acute respiratory distress syndrome, which potentially could be related to PRT-induced platelet activation [4]. Indeed, platelets have been implicated in experimental transfusion-related acute lung injury (TRALI) as critical mediators, along with neutrophils, of lung barrier disruption [5–7]. Platelets are armed with a variety of mediators that could potentially injure the lung barrier, and platelets may also “partner” with other leukocytes (neutrophils, monocytes) to enhance inflammatory responses [8].

In this study, we tested a PRT based on the addition of a photosensitizing agent, (riboflavin, vitamin B_2_), to human platelet units followed by ultraviolet light illumination [9]. This process creates chromosomal strand breakage, which effectively renders a variety of pathogens inactive. We tested whether Mirasol PRT activates human platelets and whether these platelets, when transfused into immunodeficient mice, induces lung injury. We also tested whether platelets treated with Mirasol PRT accumulate in the lungs after transfusion. Our results indicate that Mirasol PRT produces platelet activation during storage, but the platelet activation does not lead to greater retention in the lungs or the development of lung injury.

## Materials and Methods

### Mice

NOD/SCID mice (Jackson Laboratories) at 6–10 weeks of age were used for all experiments. All mice were housed in pathogen-free conditions.

### Ethics Statement

Blood was purchased from the Blood Centers of the Pacific (San Francisco, CA) and the authors had no contact with the blood donors. Therefore, this study does not involve human subjects research. The study was carried out in strict accordance with the recommendations in the Guide for the Care and Use of Laboratory Animals of the National Institutes of Health. The protocol was approved by the Institutional Animal Care and Use Committee at the University of California, San Francisco (Protocol #AN099492). All surgery was performed under ketamine and xylazine anesthesia, and all efforts were made to minimize suffering.

### Human platelet collection and Mirasol PRT treatment

Human, apheresis double dose platelet concentrates were collected from the Blood Centers of the Pacific (San Francisco, CA) in the afternoon in ACDA using a Trima collection device (Terumo BCT, USA), with a target yield of 5.5–7.0 x 10^11^ platelets/unit. Platelets were stored overnight on platelet agitators in a temperature-controlled room. The following morning, half of the volume of each product was transferred to a Mirasol Illumination Bag (MIB) while the other half remained in the collection bag. Thirty-five (35) mL of 500 μM riboflavin solution was added to the test units for treatment with the Mirasol system, and these products received a UV energy dose of 6.24 J/mL. To mimic the dilution of the test products with riboflavin, 35 mL of normal saline was added to the untreated control products. All products were stored under standard blood banking conditions until the time of analysis.

### Mouse model and acute lung injury measurements

NOD/SCID mice were anesthetized with ketamine and xylazine and human platelets were transfused via the jugular vein. The following conditions were tested: 10^8^ platelets (untreated control vs. Mirasol PRT) on days 1 and 5 of storage, 10^9^ platelets (control vs. Mirasol PRT) on days 1 and 5 of storage, and LPS (*Escherichia coli* O55:B5; Sigma-Aldrich) priming (0.1 mg/kg, i.p., 24 hours prior) followed by 10^8^ platelets (control vs. Mirasol PRT) on days 1 and 5 of storage, and LPS priming (0.1 mg/kg, i.p., 24 hours prior) followed by 10^9^ platelets (control vs. Mirasol PRT) on days 1 and 5 of storage. We also test thrombin-receptor activating peptide (TRAP) stimulated platelets by incubating platelets with TRAP (50 μM) for 10 minutes prior to transfusion. At 4 hours after transfusion, mice were euthanized and the blood and lungs collected for acute lung injury measurements. We measured extravascular lung water using the gravimetric method and lung vascular permeability by injecting mice with ^125^I-albumin (Iso-Tex Diagnostics) in the jugular vein at the time of transfusion and measuring the extravasation of the radiolabeled albumin into the bloodless lung using a gamma counter (Packard 5000 Series) [7].

### TRALI model

A two-event TRALI model was used, as previously described [6]. Briefly, after being primed with LPS (0.1 mg/kg, i.p.) for 24 hours, NOD/SCID mice were challenged with a mouse MHC I mAb (ATCC 34-1-2S; H2K^d^; IgG_2a_, κ; 1.0 mg/kg) vs. isotype control mAb (BD Biosciences; Cat. 553453) injected into the jugular vein, and euthanized after 2 hours. Extravascular lung water and lung vascular permeability were measured.

### Flow cytometry

Reagents were purchased from BD Biosciences. Platelets were identified by human glycoprotein IIb surface staining (CD41a-PE; IgG1, κ; Cat. 555467). Platelet activation was determined by P-selectin expression (CD62P-APC; IgG1, κ; Cat. 550888) on CD41+ events. All antibodies were used at optimum concentration for maximum fluorescence with minimum nonspecific binding, as described by the manufacturer. Platelets (100 μL) were prepared for flow cytometric analysis by adding 20μL of CD41a or CD62P antibodies and gently mixing. The samples were incubated for 60 minutes, and then diluted with 1 mL of BD PhosFlow Lyse/Fix Buffer (Cat. 558049) to fix, lyse the red cells, and inhibit further activation of the platelets. Incubations were carried out at room temperature (20 to 22°C) in the dark, and all samples were run in duplicate. Fifty thousand platelets were analyzed, and the results represent the means of duplicate samples. Samples were analyzed, within 2 hours of collection with a LSRII/Fortessa flow cytometer (BD Biosciences). The platelet population was identified from its light scatter characteristics and confirmed using the anti-CD41a mAb. All data were analyzed using FlowJo software (TreeStar).

### Lung intravital microscopy

We used two-photon microscopy adapted for the ventilated mouse lung to track human platelets in the lung after transfusion [10]. Briefly, human platelets (control vs. Mirasol PRT) on storage days 1 and 5 were fluorescently labeled using a CellTrace CFSE Cell Proliferation kit (Life Technologies) according the manufacturer’s instructions and washed prior to transfusion (10^9^) into anesthetized, mechanically ventilated NOD/SCID mice ± LPS priming. CFSE-labeled platelets were excited with a two-photon laser tuned to 820 nm. We captured a 210 μm x 210 μm (0.05 mm^2^) area of lung at 10 μm z-depth and images were captured every 1 second for 10 minutes. Images were analyzed with Imaris 7.6.1 software (Bitplane), and CFSE+ events were counted at 10 minutes post-injection in the treatment groups.

### Statistical analysis

Results are reported as mean ± SD. To determine significance, 2-tailed Student’s *t* and ANOVA tests were used as appropriate (GraphPad PRISM version 5.0). *P* values of less than or equal to 0.05 were deemed to be significant.

## Results

### Mirasol PRT treatment of human platelets increases surface expression of P-selectin

**Fig 1:**
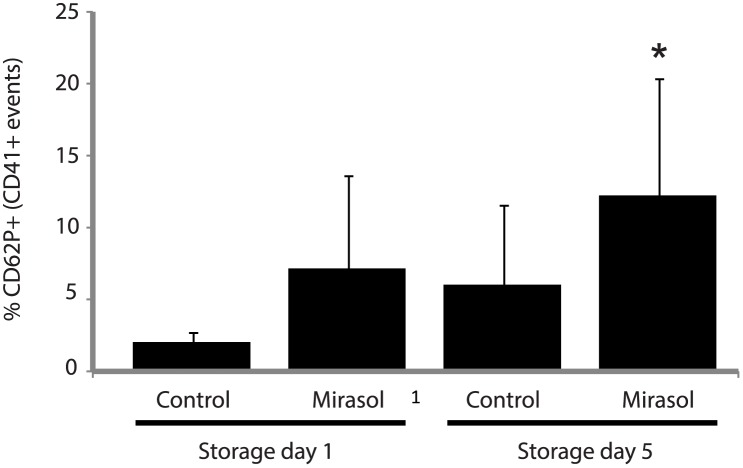
Platelet activation in control and Mirasol PRT-treated human platelets. P-selectin (CD62P) expression by human platelets on storage days 1 and 5 ± Mirasol PRT. *p<0.05 vs. day 5 control. n = 4–6 per group. Mean ± SD.

We tested whether Mirasol PRT-treatment of human platelets induces activation by testing for surface P-selectin (CD62P) expression by flow cytometry. Platelets were identified by CD41a expression and reported as the %CD62^+^/CD41^+^ events. There was trend (p = 0.16) for increased P-selectin expression in the Mirasol PRT group on day 1 of storage (Fig 1). On day 5 of storage, platelets treated with Mirasol PRT had increased expression of CD62P (Fig 1).

### NOD/SCID mice develop lung injury in a two-event model of TRALI

**Fig 2:**
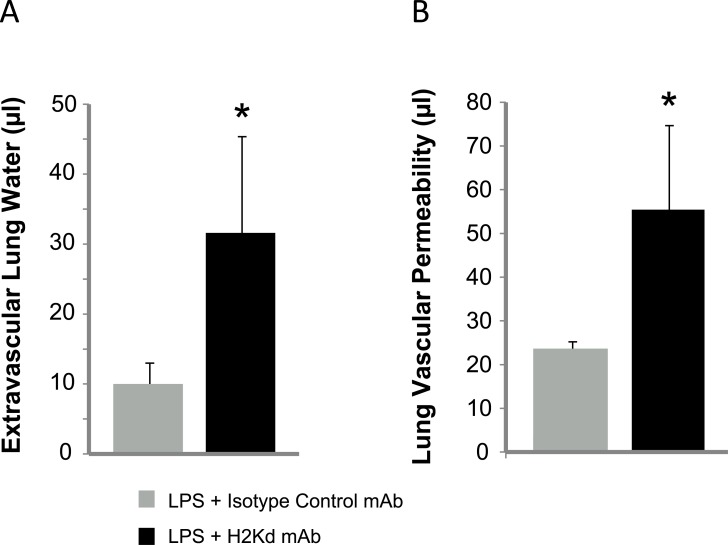
Lung injury in LPS-primed NOD/SCID mice challenged with H-2Kd mAb. **A.** Extravascular lung water in LPS-primed (0.1 mg/kg i.p.) NOD/SCID mice challenged with H2Kd mAb (1 mg/kg, i.v.) vs. isotype control mAb. **B.** Lung vascular permeability to ^125^I-albumin (i.v.) in LPS-primed (0.1 mg/kg i.p.) NOD/SCID mice challenged with H2Kd mAb (1 mg/kg, i.v.) vs. isotype control mAb. *p<0.05 vs. LPS + isotype control mAb groups. Mean ± SD. n = 5 per group.

We first tested if NOD/SCID mice were able to develop lung injury in a two-event model of TRALI. NOD/SCID mice have the H-2Kd haplotype and thus should be susceptible to cognate H2Kd mAb. NOD/SCID mice also have normal numbers of circulating neutrophils and platelets, which are critical effector cells in producing lung injury. NOD/SCID mice were challenged with 1 mg/kg of H2Kd mAb vs. isotype control mAb and the H2Kd mAb group had significant increases in extravascular lung water and lung vascular permeability, indicative of acute lung injury (Fig 2). We did not observe any mortality at 2 hours post-Ab injection.

### Transfusion of Mirasol PRT-treated platelets or control platelets does not produce acute lung injury

**Fig 3:**
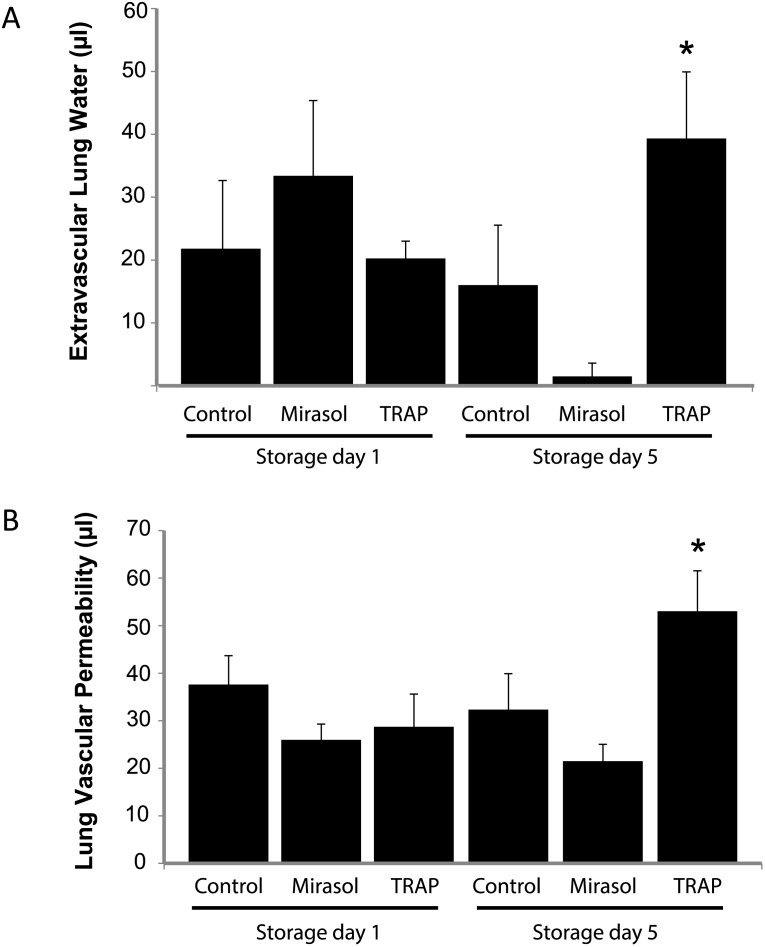
Lung injury in control, Mirasol PRT, and TRAP-treated human platelets transfused into NOD/SCID mice. **A.** Extravascular lung water in NOD/SCID mice transfused with 10^9^ human platelets (control vs. Mirasol PRT vs. TRAP-activated) on day 1 or day 5 of storage. **B.** Lung vascular permeability to ^125^I-albumin (i.v.) in NOD/SCID mice transfused with 10^9^ human platelets (control vs. Mirasol PRT vs. TRAP-activated) on day 1 or day 5 of storage. *p<0.05 vs. day 5 control and Mirasol PRT groups. TRAP = thrombin receptor activating peptide (50 μM). Mean ± SD. n = 5 per group.

Having determined that NOD/SCID mice were capable of developing TRALI, we next challenged mice with control vs. Mirasol PRT platelet transfusions on storage days 1 and 5. We tested two doses of platelets: 10^8^ and 10^9^ per mouse. As a positive control, we added TRAP (50 μM) to untreated platelets 10 minutes prior to transfusion. In Fig 3, transfusion of TRAP-stimulated platelets (10^9^) on storage day 5 produced significant increases in both extravascular lung water and lung vascular permeability, similar to that observed in the H2Kd mAb group in Fig 2. We did not detect any differences in lung injury measurements in control or Mirasol PRT platelets transfused on storage days 1 or 5 (Fig 3), and in general, lung injury measurements after control or Mirasol PRT platelet transfusions were not significantly different than the LPS + isotype control mAb group in Fig 2. We also did not detect significant increases in lung injury in either the control or Mirasol PRT platelets when transfused at 10^8^ platelets/mouse (data not shown).

### Transfusion of Mirasol PRT-treated platelets or control platelets does not produce acute lung injury in a two-event model with LPS

**Fig 4:**
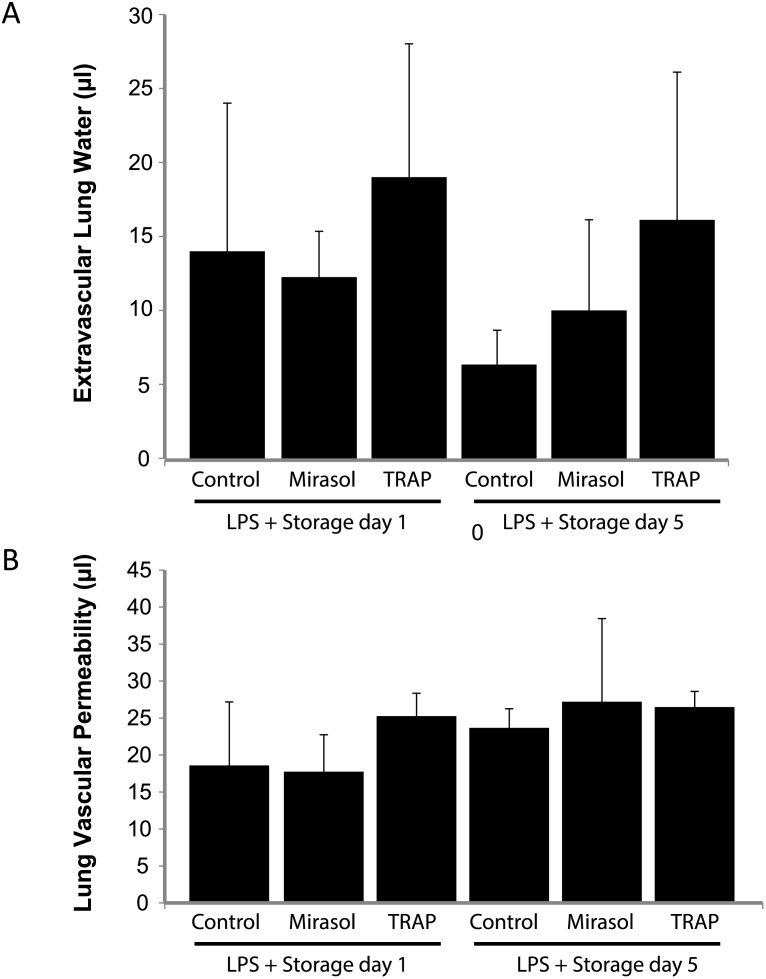
Lung injury in control, Mirasol PRT, and TRAP-treated human platelets transfused into LPS-primed NOD/SCID mice. **A.** Extravascular lung water in LPS-primed (0.1 mg/kg i.p.) NOD/SCID mice transfused with 10^8^ human platelets (control vs. Mirasol PRT vs. TRAP-activated) on day 1 or day 5 of storage. **B.** Lung vascular permeability to ^125^I-albumin (i.v.) in LPS-primed (0.1 mg/kg i.p.) NOD/SCID mice transfused with 10^8^ human platelets (control vs. Mirasol PRT vs. TRAP-activated) on day 1 or day 5 of storage. Mean ± SD. n = 5 per group.

Since experimental and clinical transfusion-related acute lung injury (TRALI) involves a two-event hypothesis in which immune priming factors in the transfusion recipient are necessary to produce lung injury [6], we next challenged mice with intraperitoneal LPS (0.1 mg/kg) 24 hours prior to human platelet transfusion. In Fig 4, LPS priming did not augment lung injury in any of the groups (control, Mirasol PRT, TRAP) compared to lung injury measurements in Fig 3. Lung injury measurements in control and Mirasol PRT groups were similar to measurements in the LPS + isotype control mAb group in Fig 2.

### Transfused Mirasol PRT-treated platelets are not differentially retained in the lungs vs. control platelets

**Fig 5:**
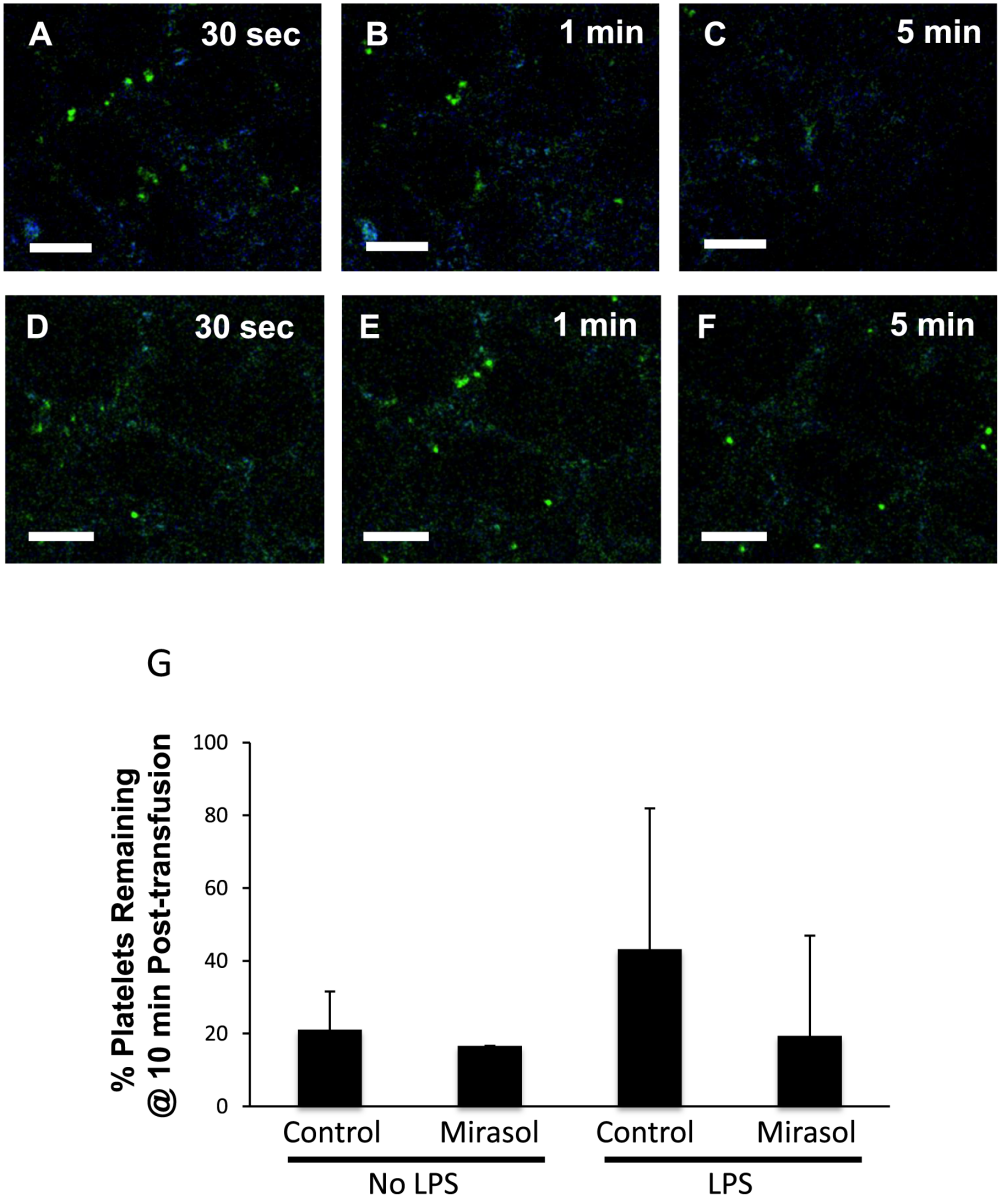
Real-time lung imaging of human platelets in the lungs of NOD/SCID mice. Lung intravital microscopy in unprimed NOD/SCID mice transfused with CFSE-labeled control (A-C) or Mirasol-PRT platelets (D-F) both on storage day 5 (10^9^ platelets). Images were captured every second for 10 minutes and screenshots are shown from 30 sec, 1 min, and 5 min post-transfusion. Scale bar = 50 μm. (G) %Platelets remaining in the imaged lung @ 10 minutes post-transfusion (# at 10 min/maximum #) in NOD/SCID mice ± LPS priming + Control vs. Mirasol PRT-treated platelets.

We next tested whether control or Mirasol PRT platelets on storage days 1 or 5 are differentially retained in the pulmonary circulation after transfusion. We used two-photon intravital lung microscopy and CFSE-labeled platelets transfused via the jugular vein and captured images every second for 10 minutes. In Fig 5A–5C, a representative example is shown in which we transfused 10^9^ CFSE-labeled control platelets (day 5 of storage) into unprimed NOD/SCID mice and screenshots are shown at 30 sec (A), 1min (B), and 5min (C) post-transfusion (see also S1 Movie). A representative example is shown in Fig 5D–5F from Mirasol PRT-treated platelets (10^9^, day 5 of storage) transfused into unprimed NOD/SCID mice (see also S2 Movie). The blue color in Fig 5 and the S1 and S2 Movies is collagen’s second harmonic generation, which aids in visualizing the alveolar structure of the lung. For our analysis, we counted the number of platelets remaining at 10 minutes post-transfusion and divided by the maximum number of platelets imaged during any frame in the 10 minute movie. We did not detect any differences in day 1 and day 5 platelet retention and therefore combined these groups in our analysis. In general, the majority of transfused platelets had exited the visualized lung microcirculation at 10 minutes post-transfusion, but 20–40% remained (Fig 5G). However, we did not detect any statistically significant differences in the retention rate of platelets between the groups (Fig 5G).

## Discussion

The major findings from this study are (1) Mirasol PRT treatment of human platelets produces platelet activation that is storage-dependent, (2) NOD/SCID, despite major deficits in cellular and humoral immunity, are susceptible to a two-event model of TRALI, (3) transfusion of control or Mirasol PRT human platelets into NOD/SCID mice, even at high doses and with LPS priming, does not produce significant acute lung injury or differential retention in the lung microcirculation.

PRT has the potential to improve the safety of the blood supply and may be especially useful in resource-limited settings, such as the military theatre, where sophisticated molecular screening for blood-borne pathogens is not feasible. However, it is imperative that non-infectious transfusion complications not be increased by the transfusion of PRT-treated products. One such concern is the development of acute lung injury (ALI) or the acute respiratory distress syndrome (ARDS), which in one clinical study was increased in the psoralen-based PRT-treated platelet product group [4]. The potential association of PRT-treated products with ALI is a serious safety concern, and in this study we designed pre-clinical experiments to determine whether a specific form of PRT, Mirasol PRT, applied to human platelets led to the development of lung injury.

We detected that human platelets treated with Mirasol PRT have evidence of activation that was storage dependent. Others have also reported similar findings on platelet activation with Mirasol PRT [11]. P-selectin expression, in particular, is not just a marker of activated platelets, but could mediate critical interactions with leukocytes that express its ligand, PSGL-1, and therefore could drive inflammatory responses after transfusion [12]. However, despite evidence of platelet activation with Mirasol PRT, when these platelets were transfused into immunodeficient mice and tracked using two-photon intravital lung microscopy, we did not detect an increased retention rate compared to control platelets. Further, transfusion of Mirasol PRT-treated platelets did not result in lung injury even with recipient priming with LPS and with the delivery of high concentrations of platelets. We tested two doses of platelet transfusions, 10^8^ and 10^9^, and the latter dose represents approximately >50% of the total native mouse platelet circulating pool, which is a significant challenge.

NOD/SCID mice were chosen as the transfusion recipients in this study, since we were transfusing human platelets and the defects in humoral and cellular immunity in these mice make them appropriate recipients [13]. We tested whether the residual components of the immune system of NOD/SCID mice (neutrophils, NK cells, platelets) would be sufficient to produce lung injury. We chose a neutrophil- and platelet-dependent model of antibody-mediated transfusion-related acute lung injury (TRALI), and indeed the NOD/SCID mice developed lung injury, although the magnitude of injury is less than in wild-type mice [6]. This is likely due to the mixed MHC Class I background of these mice (less cognate H-2K^d^ antigen) compared with BALB/c wild-type mice (pure H-2K^d^). NOD/SCID mice also developed lung injury when challenged with TRAP-activated platelets, so we conclude that lung injury can be studied in this mouse model. Others have reported that SCID mice, which are similar, but less immunodeficient than NOD/SCID mice, are also susceptible to TRALI [14, 15].

Our results are consistent with recently published results using Mirasol PRT-treated human platelets transfused into SCID recipients, which did not result in either increased lung accumulation or the development of lung injury [15]. Only when the PRT treatment (illumination) was repeated multiple times on the same platelet product was there an increase in platelet accumulation in the lungs [15]. Notably, when human platelets were treated with high-dose UVB irradiation alone and transfused into LPS-primed SCID mice, platelet accumulation in the lungs and lung injury were observed [16, 17]. However, the dose of UVB irradiation that was studied is not used in commercial PRT systems, and therefore these results have uncertain clinical relevance.

Our study has limitations. Our studies do not directly compare to the transfusion conditions in humans. Although the blood collection and Mirasol PRT treatment were done using clinical protocols, we transfused the platelets into immunodeficient mice with normal platelet counts, which does not mimic the majority of transfusion recipients. Also, our study was limited to 4 hours after transfusion, and therefore longer-term complications of platelet transfusion were not studied. However, lung injury developed within 4 hours in both the TRALI model and with TRAP-activated platelets, so we believe it is unlikely that delayed lung injury was missed.

In conclusion, although Mirasol PRT-treatment of human platelets produces storage-dependent platelet activation, when these platelets were transfused at a high dose into LPS-primed mice, we did not observe increased sequestration in the lung microcirculation or the development of lung injury. These data do not support an adverse pulmonary reaction to the transfusion of PRT-treated platelets and is important safety data for the clinical development of PRT technology.

## Supporting Information

S1 Movie Two photon lung intravital imaging in an unprimed NOD/SCID mouse transfused with CFSE-labeled control platelets (10^9^, storage day 5) and imaged every second for 5 minutes. x, y, z dimensions 210 μm, 210 μm, and 10 μm, respectively. Scale bar = 50 μm.
(AVI)

S2 Movie Two photon lung intravital imaging in an unprimed NOD/SCID mouse transfused with CFSE-labeled Mirasol-PRT platelets (10^9^, storage day 5) and imaged every second for 5 minutes. x, y, z dimensions 210 μm, 210 μm, and 10 μm, respectively. Scale bar = 50 μm.
(AVI)
